# Gout Treated with Belladonna and Naphtha

**Published:** 1845-11

**Authors:** Alexander Ure


*Gout treated with Belladonna and Naphtha.* By Alexander Ure, Esq.—J. P., æt. 45, moderate and regular in his habits, but of late rather sedentary, was suddenly attacked for the second time with the gout, which commenced in the ball of the great toe of left foot. An ointment composed of one part ext. belladonna, and eight of cerate, was ordered to be applied with gentle friction over the affected part, and five grains each of blue pill and colocynth to be taken at bed time. By this treatment the pain was dulled, but the toe remained swollen and of a dusky red. He was then ordered to use mineral naphtha as an embrocation. Derived direct benefit from penciling over the part with naphtha; the pain, redness, and swelling were wholly removed. He recovered. This case is adduced in order to show the efficacy of rectified coal naphtha in the subacute form of inflammation.—*Med. Gazette.—Braithwait's Retrospect.*

